# *Mycobacterium tuberculosis* ClpC1 N-Terminal Domain Is Dispensable for Adaptor Protein-Dependent Allosteric Regulation

**DOI:** 10.3390/ijms19113651

**Published:** 2018-11-19

**Authors:** Justin D. Marsee, Amy Ridings, Tao Yu, Justin M. Miller

**Affiliations:** 1 Department of Chemistry, Middle Tennessee State University, 1301 East Main Street, Murfreesboro, TN 37132, USA; jdm2am@mtmail.mtsu.edu; 2 Department of Chemistry, Tennessee Tech University, 1 William L Jones Drive, Cookeville, TN 38505, USA; aridings42@students.tntech.edu

**Keywords:** Clp/Hsp100 proteins, ClpC1, *Mycobacterium tuberculosis*, ClpS, adaptor proteins, ATP-dependent protease

## Abstract

ClpC1 hexamers couple the energy of ATP hydrolysis to unfold and, subsequently, translocate specific protein substrates into the associated ClpP protease. Substrate recognition by ATPases associated with various cellular activities (AAA+) proteases is driven by the ATPase component, which selectively determines protein substrates to be degraded. The specificity of these unfoldases for protein substrates is often controlled by an adaptor protein with examples that include MecA regulation of *Bacillus subtilis* ClpC or ClpS-mediated control of *Escherichia coli* ClpA. No adaptor protein-mediated control has been reported for mycobacterial ClpC1. Using pulldown and stopped-flow fluorescence methods, we report data demonstrating that *Mycobacterium tuberculosis* ClpC1 catalyzed unfolding of an SsrA-tagged protein is negatively impacted by association with the ClpS adaptor protein. Our data indicate that ClpS-dependent inhibition of ClpC1 catalyzed SsrA-dependent protein unfolding does not require the ClpC1 N-terminal domain but instead requires the presence of an interaction surface located in the ClpC1 Middle Domain. Taken together, our results demonstrate for the first time that mycobacterial ClpC1 is subject to adaptor protein-mediated regulation in vitro.

## 1. Introduction

ATP-dependent proteases represent a family of molecular machines responsible for the regulated turnover of misfolded, aggregated, or degradation-tagged cellular proteins [1,2,3,4]. In mycobacteria, the regulated removal of protein substrates in the cytoplasm is mediated by at least four different proteolytic complexes that are broadly divided into two groups that include the bacterial-like proteases (FtsH, Lon, ClpXP and ClpC1P) and the eukaryotic-like proteasome [5,6]. These proteases share a common architecture in which a ring-shaped AAA+ (ATPases Associated with various cellular Activities) ATPase associates with one or both ends of a barrel-shaped peptidase that contains active sites inaccessible to bulk solvent [3,5]. In the active state, the hexameric ATPase couples the energy of ATP hydrolysis to unfold and thread protein substrates into the associated protease for degradation.

Substrate recognition by AAA+ proteases is driven by the ATPase component, which functions as a gate-keeper to selectively determine protein substrates to be degraded. However, the specificity of AAA+ unfoldases for protein substrates is often modulated by an associated adaptor protein. For example, *Escherichia coli* (*E. coli*) ClpA specifically recognizes the SsrA degradation sequence, which is a C-terminal degradation tag that is co-translationally added by the tmRNA tagging system to nascent polypeptide chains of stalled ribosomes [7]. ClpA has been reported to bind the SsrA sequence with an affinity equal to ~200 nM, which drives specific degradation of SsrA-tagged proteins by the ClpAP complex [8]. Association with the ClpS adaptor protein results in a substantial decrease in the affinity of ClpA towards SsrA-tagged proteins such that SsrA-tagged substrates are not degraded by the ClpAPS complex [9,10,11]. Deletion of the N-terminal domain of ClpA reverses this observation such that ClpAP catalyzed degradation of an SsrA-tagged Green Fluorescent protein occurs independent of *E. coli* ClpS [9]. Complimentary work has demonstrated that a single ClpS molecule associates with ClpA hexamers with an affinity of ~40 nM but that an additional one to two ClpS molecules may associate with a decreased affinity equal to >700 nM [11,12]. Taken together, saturation of ClpS binding to ClpA hexamers as indicated by a 6:1 ratio of ClpS:ClpA_6_ is not necessary for significant control of ClpA function but an intact N-terminal domain (NTD) is required for ClpS-dependent inhibition of ClpAP catalyzed degradation of SsrA-tagged protein substrates.

Until recently, ClpC protein function was widely thought to depend on adaptor protein association. *Bacillus subtilis* (*B. subtilis*) ClpC oligomerization and subsequent chaperone activity was previously reported to depend on the association of the MecA adaptor protein [13,14]. High-resolution structures for *Staphylococcus aureus* (*S. aureus*) ClpC in the presence and absence of MecA have recently revealed a more complicated picture. Association of the MecA adaptor protein with *S. aureus* ClpC promotes formation of enzymatically active ClpC hexamers via transition from an inactive helical assembly [15]. *B. subtilis* ClpCP can also bind and degrade phosphoarginine substrates independent of any adaptor protein [16], thereby demonstrating that MecA association is not always required for ClpCP function as once thought. In contrast, ClpC proteins from cyanobacteria and actinobacteria are not widely known to possess chaperone activities that are adaptor protein-dependent [17,18,19,20,21]. All available data describing actinobacterial ClpC protein function have been collected using *Mycobacterium tuberculosis* (*M. tuberculosis*) ClpC1 (ClpC2 lacks identifiable motifs associated with ATPase activity) and demonstrate adaptor protein-independent activity. However, the protein degradation activity of *Synechoccus elongatus* ClpCP3/R is regulated by two ClpS isoforms, ClpS1 and ClpS2, where ClpS1 promotes the binding of N-degron protein substrates bearing N-terminal Phe and Tyr residues and ClpS2 blocks degradation of α-casein substrates [18]. No data has been reported regarding adaptor protein-dependent regulation of Clp protease complexes in actinobacteria.

Given the close phylogenetic relationship between mycobacterial and cyanobacterial ClpC proteins [22], it is likely that the former may be subject to ClpS-mediated control. However, it is currently unclear whether mycobacterial ClpC1 is subject to regulation by any adaptor protein. For this reason, we set out to determine the functional relationship between *M. tuberculosis* H37Rv ClpC1 (Accession Number: *Rv3596c*) and ClpS (Accession Number: *Rv1331*). That is to say, if a physical ClpC1:ClpS interaction occurs, does it impact ClpC1 function? Here, we demonstrate that a physical association occurs to form a stable complex detectable by pulldown methods. From stopped-flow fluorescence experiments reporting on ClpC1 catalyzed unfolding of an SsrA-tagged fluorescent protein, we report a [ClpS]-dependent two-state transition between a fully catalytic state and a partially-inhibited ClpC1 state. Our data suggest that specific residues located in the Middle Domain are necessary for allosteric control by ClpS independent of the ClpC1-NTD. These results demonstrate that ClpS allosterically impacts ClpC1 catalyzed unfolding of an SsrA-tagged protein and concurrently represent the first report of adaptor protein-mediated regulation of a mycobacterial ClpC protein.

## 2. Results

### 2.1. Dependence of Apparent Unfolding Rate Constant on [ClpS]

**Figure 1:**
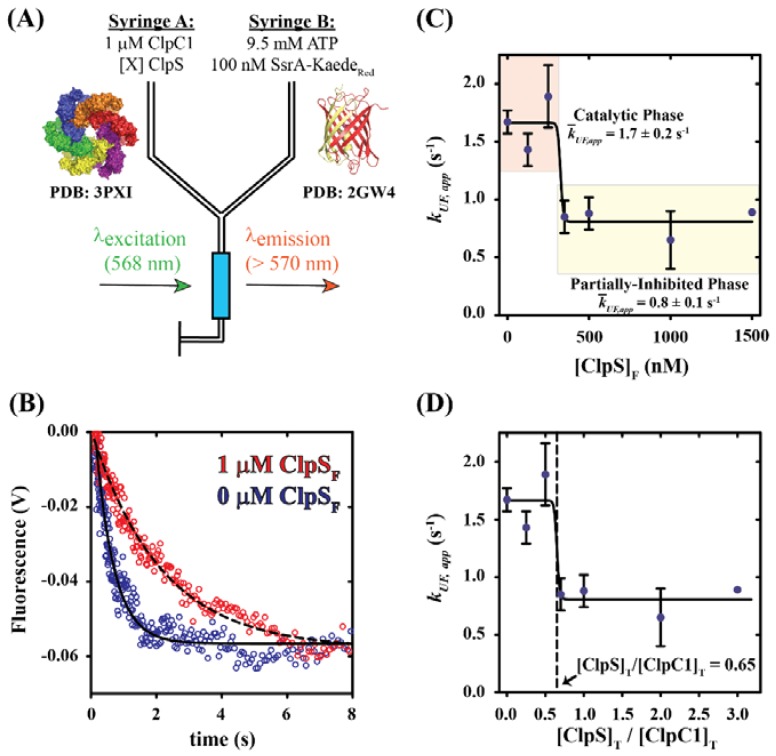
Examining the impact of [ClpS] on ClpC1 catalyzed unfolding of an SsrA-tagged protein. (**A**) Schematic representation of stopped-flow fluorescence protein unfolding experiments. Syringe A contains the indicated reagents, 1 µM ClpC1 and varied concentrations of ClpS (indicated in text). Syringe B contains 9.5 mM ATP to fuel protein unfolding and 100 nM photoactivated Kaede bearing a C-terminal SsrA-degradation tag (SsrA-Kaede_Red_). Fluorescence is observed using an excitation wavelength equal to 568 nm and emissions are observed above 570 nm with a 570-nm-long pass filter. Upon mixing, the concentrations are two-fold lower than in the preincubation syringe. (**B**) Representative fluorescence time courses for ClpC1 catalyzed SsrA-Kaede_Red_ unfolding. Time courses represent 1 µM ClpC1 incubated with (Red Circles) or without (Blue Circles) 2 µM ClpS prior to mixing with 9.5 mM ATP and 100 nM SsrA-Kaede_Red_. The dashed and solid lines represent nonlinear least squares (NLLS) fits using a single-exponential function for time courses collected in the presence or absence of ClpS, respectively. All apparent unfolding rate constants are summarized in Table 1. (**C**) Dependence of the apparent unfolding rate constant, *k_UF,app_*, on [ClpS]_F_, where [ClpS]_F_ represents the final mixing concentration of ClpS. Average unfolding rate constants for the Catalytic and Partially-Inhibited phases are equal to 1.7 ± 0.2 and 0.8 ± 0.1 s^−1^, respectively. (**D**) Replotting the data shown in Figure 1C as *k_UF,app_* versus [ClpS]_T_/[ClpC1]_T_ indicates that the transition from Catalytic phase to Partially-Inhibited phase occurs when the ratio of total ClpS concentration to total ClpC1 monomer concentration equals 0.65. All data shown are from independent experiments and error bars indicate ± standard deviation.

To determine whether mycobacterial ClpS is a functional adaptor of ClpC1, we performed stopped-flow fluorescence experiments using a method that reports on ClpC1 catalyzed unfolding of an SsrA-tagged protein. Figure 1A illustrates the experimental design as described in Materials and Methods. In our experiments, syringe A of the stopped-flow fluorometer contains a solution of 1 µM ClpC1 incubated in the presence or absence of ClpS. Syringe B is loaded with a solution containing 9.5 mM ATP and 100 nM photoactivated SsrA-Kaede (SsrA-Kaede_Red_). *Trachyphyllia geoffroyi* (*T. geoffroyi*) Kaede belongs to a family of fluorescent proteins that are structurally homologous to the green fluorescent protein (GFP) from *Aequorea Victoria* [23]. Kaede contrasts GFP through photoactivation-dependent excitation and emission properties, where irradiation by ultraviolet-visible light (350–410 nm) causes peptide cleavage adjacent to His62 via a β-elimination reaction [23,24,25]. Cleavage by photoactivation yields Kaede_Red_, which exhibits red-shifted emissions and a chromophore structure that is distinct from the green (non-photoactivated) form [23]. In our experiments, we include Kaede_Red_ bearing a C-terminal SsrA degradation tag (SsrA-Kaede_Red_). As illustrated by Glynn and coworkers with *E. coli* ClpX, unfolding of SsrA-Kaede_Red_ irreversibly displaces an N-terminal Kaede fragment and quenches native Kaede_Red_ emissions [26]. From this, we expect that mixing the contents of the two syringes depicted in Figure 1A will result in ClpC1 catalyzed unfolding of SsrA-Kaede_Red_ and subsequent quenching of fluorescence observed at wavelengths longer than 570 nm.

Figure 1B shows representative fluorescence time courses collected by rapidly mixing the contents of syringes A and B, as schematized in Figure 1A. The representative time courses were collected with final mixing concentrations of each reactant equal to 0.5 µM ClpC1, 50 nM SsrA-Kaede_Red_ and 4.75 mM ATP in the presence (Red trace in Figure 1B) and absence (Blue trace in Figure 1B) of 1 µM ClpS. As predicted, all time courses display a time-dependent decrease in observed emissions. The magnitude of the observed change in SsrA-Kaede_Red_ emissions in this experimental design is low relative to previously reported translocation assays performed with other Clp/Hsp100 family members [27,28]. To insure that our observed emissions signal was reproducible, we repeated the experiments shown in Figure 1B six times using protein samples derived from independent preparations. As such, we are confident in asserting that the rate of ClpC1 catalyzed unfolding of SsrA-Kaede_Red_ is dependent on [ClpS]. Nonlinear least squares (NLLS) analysis of each time course shown in Figure 1B using a single-exponential function yields an apparent unfolding rate constant, *k_UF,app_*, equal to 1.7 ± 0.1 or 0.7 ± 0.3 s^−1^ for conditions including 0 or 1 µM ClpS_F_ (final mixing [ClpS]), respectively. Our observation of a nearly three-fold decrease in the apparent unfolding rate constant for conditions lacking ClpS relative to conditions with [ClpS]_F_ in excess lead us to conclude that *M. tuberculosis* ClpC1 is inhibited by ClpS.

We next examined the ClpS concentration dependence of the apparent unfolding rate constant describing ClpC1 catalyzed the protein unfolding. Stopped-flow fluorescence experiments were performed as schematized in Figure 1A by varying the [ClpS] in syringe A. Time courses were collected at final mixing concentrations of ClpS equal to 0, 125, 250, 350, 500, 1000 and 1500 nM. Each data set was subjected to NLLS analysis using a single-exponential function to determine the apparent rate constant (Table 1). A plot of the apparent unfolding rate constant, *k_UF,app_*, versus the final mixing concentration of ClpS, [ClpS]_F_, displays a pronounced dependence on the molar concentration of ClpS_F_ (Figure 1C). NLLS analysis of the data shown in Figure 1C using a Hill function (Equation (1)) suggests an affinity describing the ClpS:ClpC1 interaction as ~325 nM under the final mixing conditions examined here. Due to the steep slope, our analysis was unable to accurately estimate the Hill coefficient.

The maximum binding stoichiometry can be determined from the breakpoint in a plot of the degree of binding versus the ratio of the concentrations of total ligand to total macromolecule, [X]_T_/[M]_T_ [29]. Since ClpC1 is incubated with ClpS prior to mixing with ATP and SsrA-Kaede_Red_ in our experiments, we expect that the resulting apparent rate constants describing the [ClpS]_F_-dependence of SsrA-tagged protein unfolding by ClpC1 are proportional to the degree of binding. Figure 1D shows a plot of the apparent unfolding rate constant versus the ratio of total ClpS_F_ concentration to ClpC1 monomer concentration. This plot yields a curve with an inflection point equal to 0.65. An inflection point of less than unity strongly suggests a deviation from a 1:1 binding stoichiometry between ClpS and ClpC1. Calculation of the ratio of [ClpC1]_T_ to [ClpS]_T_, instead of [ClpS]_T_:[ClpC1]_T_, using monomeric terms yields an estimate of 1–2 ClpC1 molecules associated per ClpS monomer (1/0.65 [ClpS]_F_/[ClpC1]_T_) = 1.54 ([ClpC1]_T_/[ClpS]_T_)). Given that the data presented in Figure 1C,D are not hyperbolic, this observation likely indicates the average binding stoichiometry to be two ClpC1 subunits per single ClpS molecule.

### 2.2. N-Terminal Domain of ClpC1 Is Dispensable for ClpS-Mediated Inhibition of Protein Unfolding

**Figure 2:**
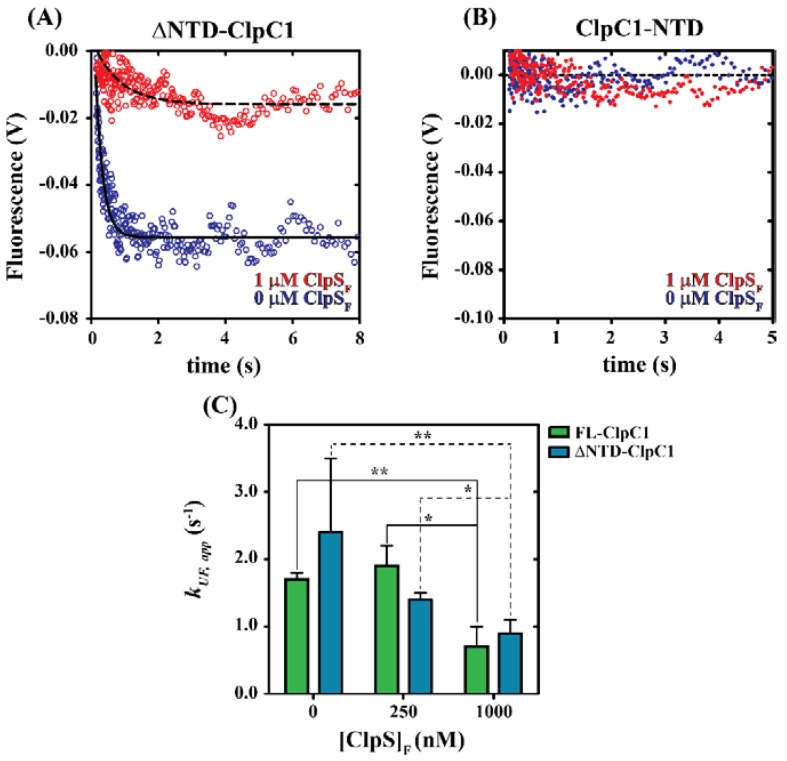
The ClpC1 N-terminal domain is dispensable for protein unfolding. (**A**) Representative fluorescence time courses for ∆NTD-ClpC1 catalyzed SsrA-Kaede_Red_ unfolding. Time courses represent 1 μM ∆NTD-ClpC1 incubated with (Red Circles) or without (Blue Circles) 2 µM ClpS prior to mixing with 9.5 mM ATP and 100 nM SsrA-Kaede_Red_. The dashed and solid lines represent NLLS fits using a single-exponential function for time courses collected in the presence or absence of ClpS, respectively. All apparent unfolding rate constants are summarized in Table 1. (**B**) Representative fluorescence time courses for ClpC1-NTD catalyzed SsrA-Kaede_Red_ unfolding with coloring identical to Figure 1A. No significant unfolding is observed; all data points are observed to fluctuate about the baseline. (**C**) Comparison of the apparent unfolding rate constants observed for full-length ClpC1 (Green Bars) and ∆NTD-ClpC1 (Blue Bars) in the presence of 0, 250 and 1000 nM ClpS. * *p* ≤ 0.05, ** *p* ≤ 0.001 calculated from an unpaired Student’s *t*-test (two-tailed). All data shown are from independent experiments and error bars indicate ± standard deviation.

Previous reports have indicated that the N-terminal domain of *M. tuberculosis* ClpC1 is dispensable for chaperone function [21]. Based on this, we asked; are N-terminal domain deletion variants of ClpC1 susceptible to negative regulation by ClpS similar to full-length ClpC1? To examine this question, we performed stopped-flow fluorescence experiments as described above and in Materials and Methods observing SsrA-Kaede_Red_ unfolding by a ClpC1 truncation mutant lacking N-terminal residues M1-Y145, which we term ∆NTD-ClpC1. Truncations were designed based on our previously reported primary sequence analysis [22] and the crystal structure for the *M. tuberculosis* ClpC1 N-terminal domain [30]. In our stopped-flow experiments with ∆NTD-ClpC1, the only modification made to the experimental design as depicted in Figure 1A is a substitution of ∆NTD-ClpC1 in place of full-length ClpC1. Figure 2A shows representative time courses collected by rapidly mixing the contents of syringes A and B, as schematized in Figure 1A. The final concentrations of all reactants upon mixing were 0.5 µM ∆NTD-ClpC1, 50 nM SsrA-Kaede_Red_, 4.75 mM ATP and 1 µM ClpS (where indicated). The representative time courses were collected in the presence (Red trace in Figure 2A) and absence (Blue trace in Figure 2A) of 1 µM ClpS_F_. All time courses display a time-dependent decrease in observed emissions. However, time courses collected by incubating ∆NTD-ClpC1 in the presence of 1 µM ClpS_F_ exhibit a marked decreased in the apparent amplitude, which suggests a decrease in the concentration of unfolded SsrA-Kaede_Red_. In the absence of ClpS, time courses reporting on ∆NTD-ClpC1 catalyzed unfolding of SsrA-Kaede_Red_ yield amplitudes that are similar to the same experiments performed using full-length ClpC1. Comparable experiments performed using ClpC1-NTD in place of either full-length ClpC1 or ∆NTD-ClpC1 reveal no change in fluorescence signal, consistent with the requirement for ATP hydrolysis for productive protein unfolding to occur (Figure 2B).

Each time course shown in Figure 2A was subjected to NLLS analysis using a single-exponential function to determine the apparent rate constant (Table 1). The resulting apparent unfolding rate constants, *k_UF,app_*, corresponding to both full-length ClpC1 and ∆NTD-ClpC1 are plotted in Figure 2C. In the absence or presence of 1 µM ClpS_F_, the apparent unfolding rate constant describing ∆NTD-ClpC1 catalyzed unfolding of SsrA-Kaede_Red_ is 2.4 ± 1.1 or 0.9 ± 0.2 s^−1^, respectively. Unfolding rate constants estimated in the presence of 0 or 250 nM ClpS_F_ for either full-length ClpC1 or ∆NTD-ClpC1 are statistically indistinguishable. However, comparison of means by *t*-testing demonstrate that the mean unfolding rate constants describing ClpC1 or ∆NTD-ClpC1 catalyzed protein unfolding in the presence of 0 versus 1000 nM ClpS_F_ or 250 versus 1000 nM ClpS_F_ are statistically different (Figure 2C). Taken together, our data suggest a model wherein the NTD is dispensable for ClpS-dependent allosteric inhibition of ClpC1 catalyzed unfolding of SsrA-tagged Kaede.

### 2.3. Mycobacterial ClpS Primary Sequence Analysis Reveals ClpC1-MD (Middle Domain) Binding Features

**Figure 3:**
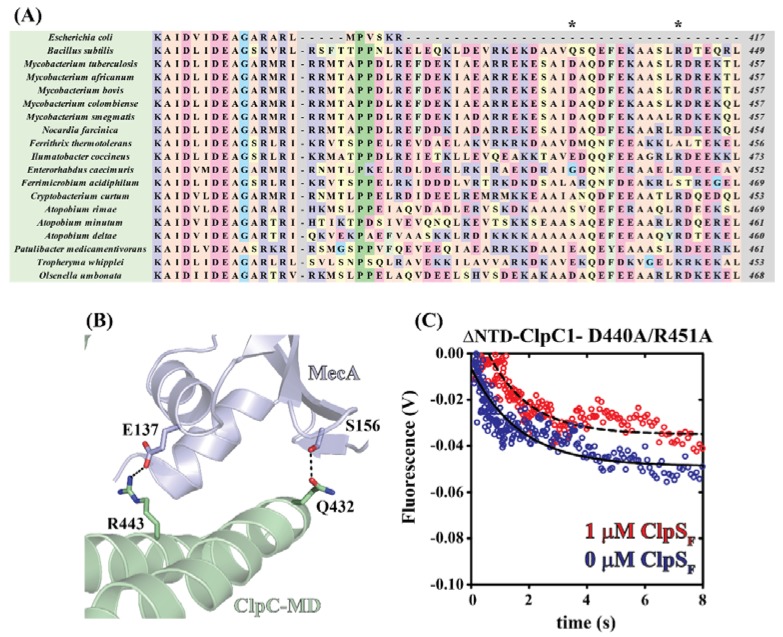
Primary sequence analyses predict the ClpC1-MD as a secondary binding surface for ClpS. (**A**,**B**) Multiple sequence alignments reveal the conservation of two residues (indicated by asterisks *) in actinobacterial ClpC1 protein sequences that are involved in complex formation between *B. subtilis* MecA and the ClpC-MD. (**C**) Representative fluorescence time courses for ∆NTD-ClpC1-D440A/R451A catalyzed SsrA-Kaede_Red_ unfolding. Time courses represent 1 µM ∆NTD-ClpC1-D440A/R451A incubated with (Red Circles) or without (Blue Circles) 2 µM ClpS prior to mixing with 9.5 mM ATP and 100 nM SsrA-Kaede_Red_. The dashed and solid lines represent NLLS fits using a single-exponential function for time courses collected in the presence or absence of ClpS, respectively. All apparent unfolding rate constants are summarized in Table 1. All structure representations in Figure 3 were prepared with the Pymol software package and Protein Databank (PDB) accession code 3PXG [31].

Previous X-ray structures for *B. subtilis* ClpC in complex with MecA indicate that complex formation involves binding interactions at the NTD and middle domain (MD) [31]. This observation led us to ask; can this prior knowledge on interactions between MecA and the *B. subtilis* ClpC-MD be used to make predictions regarding the identity of the ClpC1:ClpS interaction surface? To address this question, we utilized multiple sequence alignments to identify putative MD-ClpS interacting residues between actinobacterial ClpC and ClpS proteins (Figure 3A).

Reference to the ClpC:MecA complex structure predicts two *B. subtilis* ClpC-MD residues, R443 and Q432, to be involved in complex formation (Figure 3B) [31]. Multiple sequence alignments comparing *E. coli* ClpA and ClpC proteins demonstrates a high degree of sequence conservation among MD-containing ClpC proteins for the R443 position but lesser conservation is observed for the Q432 position (Figure 3A). From the ClpC:MecA structure, we expect that a similar interaction would be possible if a residue is conserved at the *B. subtilis* residue position 432 that harbors a side-chain with a carbonyl functional group available for hydrogen bonding to the side-chain of nearby MecA S156. Consistent with this prediction, Figure 3A illustrates that mycobacterial ClpC proteins maintain a conserved aspartate in the corresponding residue position. Thus, the mycobacterial ClpC1-MD contains primary sequence features that may support interaction with *B. subtilis* MecA.

To determine the importance of ClpC1-MD residues for ClpS-dependent inhibition of ClpC1 catalyzed protein unfolding, we further modified ∆NTD-ClpC1 to harbor D440A and R451A mutations. These mutations are based on the *B. subtilis* R443 and Q432 residue positions as discussed above. Stopped-flow fluorescence experiments performed as described in Figure 1A suggest that the ∆NTD-ClpC1-D440A/R451A mutant catalyzes unfolding of SsrA-tagged Kaede_R_ with a marked decrease in apparent unfolding rate constant regardless of [ClpS]_F_. Figure 3C reveals that time courses reporting on ∆NTD-ClpC1-D440A/R451A mutant catalyzed protein unfolding qualitatively overlay whether collected in the absence (Blue trace, Figure 3C) or presence (Red trace, Figure 3C) of 1 µM ClpS_F_. NLLS analysis of time courses shown in Figure 3C yield estimates of the unfolding rate constants equal to 0.59 ± 0.05 and 0.71 ± 0.02 s^−1^ in the presence of 0 or 1 µM ClpS_F_, respectively. Therefore, *M. tuberculosis* ClpC1-MD may represent a surface necessary for ClpS interaction.

### 2.4. Identification of ClpC1 Surfaces Involved in Complex Formation

**Figure 4:**
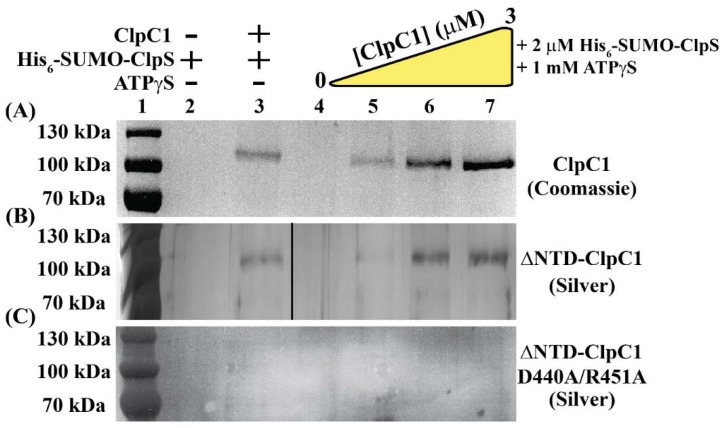
Pulldown experiments reveal a physical ClpC1:ClpS interaction. All samples were prepared by incubating 2 µM His_6_-ClpS with or without ClpC1/ATPγS. Following complex formation, His_6_-ClpS was isolated using affinity pulldown methods based on the His_6_-tag:Ni-NTA resin interaction. (**A**) *M. tuberculosis* ClpC1:ClpS protein complex formation is enhanced relative to background levels in the presence of 1 mM ATPγS. Lanes 2 and 3 were loaded with 2 µM His_6_-ClpS previously incubated with or without 2 µM ClpC1, respectively. In addition to 2 µM His_6_-SUMO-ClpS, Lanes 4–7 contained 0, 1, 2, or 3 µM ClpC1, respectively and 1 mM ATPγS. ClpC1:ClpS interactions were observed by Coomassie staining methods. (**B**) The ∆NTD-ClpC1 truncation shows diminished association with His_6_-SUMO-ClpS when compared to wild-type *M. tuberculosis* ClpC1 as indicated by the need to visual band patterns using silver staining methods. All lanes were loaded as described in Figure 4A but with ∆NTD-ClpC1 substituted in place of full-length ClpC1. (**C**) Pulldown experiments described in Figure 4A were repeated by substituting ∆NTD-ClpC1-D440A/R451A in the place of full-length ClpC1.

In order to resolve whether ClpC1:ClpS complex formation requires the ClpC1-NTD, pulldown experiments were performed using His_6_-tagged ClpS as a reporter for ClpC1 binding. Varied concentrations of ClpC1 were incubated in the presence of 2 µM His_6_-SUMO-ClpS and 1 mM ATPγS, a slowly-hydrolysable ATP analogue (Figure 4A). In this experimental design, all samples contain equimolar amounts of His_6_-SUMO-ClpS and are treated with a constant volume of Ni-NTA slurry. Thus, we expect that the amount of His_6_-SUMO-ClpS eluted from the Ni-NTA resin will remain constant across all conditions surveyed here. If His_6_-SUMO-ClpS interacts physically with ClpC1, SDS-PAGE analysis will reveal a gel band that corresponds to ClpC1 with molecular weight equal to approximately 95 kDa.

The SDS-PAGE analysis illustrated in Figure 4A confirms that a ClpC1:ClpS interaction occurs. As expected, a gel band corresponding to the molecular weight of ClpC1 is not observed by Coomassie staining when ClpC1 is not present (Figure 4A, Lanes 2 and 4). We note the presence of a ClpC1 band when ClpC1 is incubated with 2 µM His_6_-SUMO-ClpS in the presence and absence of ATPγS (Figure 4A, Lanes 3, 5–7). We are confident that this is not the result of contaminating His_6_-SUMO-ClpC1 in our ClpC1 preparation since our purification protocol includes a Ni-NTA Immobilized Metal Affinity Chromatography (IMAC) step after His_6_-SUMO-tag removal, thereby separating His_6_-SUMO-ClpC1 from cleaved ClpC1 (Section 4). We note the observation of non-specific interactions between full-length ClpC1 and Ni-NTA resin (Figure S1, Lane 1). However, the incubation of 2 µM His_6_-SUMO-ClpS with 1 mM ATPγS and 2 µM ClpC1 yields qualitatively denser gel bands (Figure S1, Lane 3), which is an observation consistent with specific binding between His_6_-SUMO-ClpS and ClpC1.

A common feature shared amongst characterized adaptor proteins is that the primary contact surfaces on the associated Clp/Hsp100 protein are located in the N-terminal domain [15,31,32]. Based on this and our stopped-flow fluorescence data presented in Figure 1 and Figure 2, we performed additional affinity pulldown experiments with ∆NTD-ClpC1. Experiments were performed by incubation of 2 µM His_6_-SUMO-ClpS with 0, 1, 2, or 3 µM ∆NTD-ClpC1 and 1 mM ATPγS (Figure 4B, Lanes 4–7). In order to resolve gel bands corresponding to ∆NTD-ClpC1 in these experiments, silver-stain methods were required for SDS-PAGE gel visualization. Similar to the identical experiments performed with full-length ClpC1, ∆NTD-ClpC1 was observed to coelute in Ni-NTA pulldown experiments under conditions including both His_6_-SUMO-ClpS and ∆NTD-ClpC1. Unlike full-length ClpC1, ∆NTD-ClpC1 was not observed to interact non-specifically with Ni-NTA resin (Figure S1). From this, we conclude that binding of ClpS to a ClpC1 construct lacking an intact N-terminal domain does occur. However, complete ablation of ClpC1:ClpS complex formation is observed when the same experiments are performed with the ∆NTD-ClpC1-D440A/R451A mutant (Figure 4C). Taken together, these data suggest that ClpC1:ClpS complex formation involves interactions between the ClpC1-MD and ClpS.

### 2.5. Molecular Dynamics Simulations Predict Unique ClpS Interface Involved in Complex Formation

**Figure 5:**
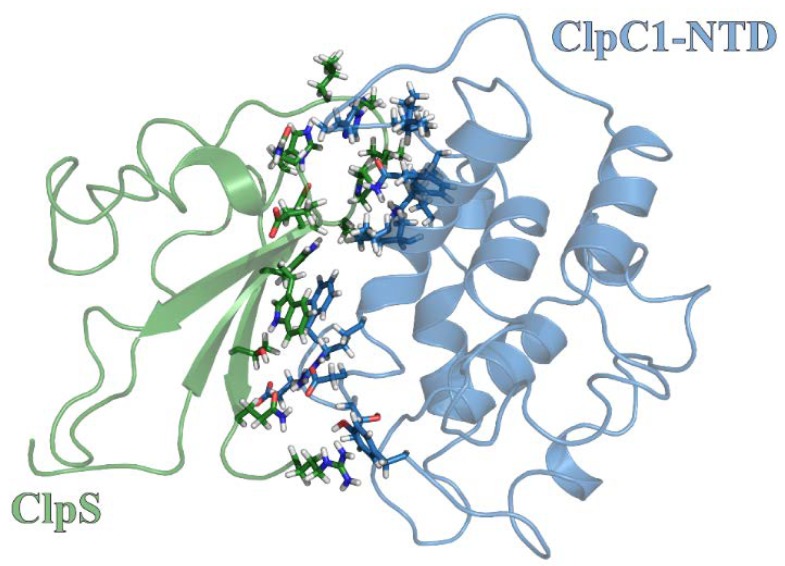
Molecular Dynamics simulations predict unique ClpS interface involved in complex formation. The snapshot of the *M. tuberculosis* ClpC1-NTD:ClpS complex obtained by the Molecular Dynamics simulation. All contact residues are highlight in stick representation. Structures for ClpC1-NTD and ClpS are shown in cartoon representation and colored as blue and green, respectively. All structure representations in Figure 5 were prepared with the Pymol software package.

Adaptor protein regulation of Hsp100 proteins commonly involves interactions with the N-terminal domain. Molecular Dynamics simulations were performed to investigate the structural arrangement necessary to drive ClpC1:ClpS assembly via the ClpC1-NTD surface. Figure 5 displays the atomistic details describing the ClpC1-NTD and ClpS binding structure obtained by molecular dynamics simulation. The contact residues in the binding interface were determined based on a separation distance less than 4.5 Å. Most contact residues were observed to be neutral but some charged residues were also involved in the binding interface. From this, we conclude that the underlying molecular driving forces that function to stabilize the ClpC1-NTD:ClpS interface represent contributions from electrostatic interactions, hydrogen bonding and hydrophobic interactions.

The interaction energies between binding pairs are listed in Table 2. From these calculated interaction energies, an overall binding interaction energy is estimated to be approximately −54 kcal/mol. However, nearly 42% of this binding energy is associated with a single interaction between Lys85 of ClpC1-NTD and Asp35 of ClpS, where the associated binding energy was estimated as −22.5 kcal/mol. Analysis of multiple sequence alignments indicates that the residue position corresponding to *M. tuberculosis* ClpC1 Lys85 is widely conserved as either a lysine or glutamine in ClpC and ClpA proteins, respectively. Separate multiple sequence alignments generated using ClpS protein sequences suggest that *M. tuberculosis* ClpS position 35 is universally conserved as an aspartic acid residue. Thus, a Lys:Asp interaction would be possible at these residue positions in most ClpC and ClpS proteins, respectively. Based on the available structural models, a similar interaction between ClpA and ClpS does not occur using the arrangement observed in our molecular dynamics model for the ClpC1-NTD in complex with ClpS.

## 3. Discussion

### ClpC1 Binding of ClpS May Involve Multiple Binding Surfaces

Recent Cryo-EM structures for the *S. aureus* ClpC:MecA [15] complex and *Saccharomyces cerevisiae* (*S. cerevisiae*) Hsp104 [33] have implicated the Middle Domain as a molecular switch in HSP100 proteins. Association of *S. aureus* ClpC with MecA induces a structural rearrangement that transitions the complex from an inactive resting state to a fully functional chaperone [15]. This switch is accompanied by a transformation from a helical assembly stabilized by head-to-head intermolecular MD contacts to the planar ring structure associated with active Clp/Hsp100 ATPases. The molecular basis for describing this process centers on the Uvr motif of the MD, a primary sequence motif characterized by a conserved motif, [E/D]ϕE, similar to the coiled-coil motifs of UvrB and UvrC (ϕ represents any aromatic residue) [34,35]. Previous data with *B. subtilis* ClpC has demonstrated Uvr motif residue F436 as critical for protein complex assembly and function [31]. The F436A mutation and outright MD deletion in *S. aureus* ClpC yields no detectable stimulation of ATPase activity in the presence of MecA [15]. Cryo-EM structures for *S. aureus* ClpC with and without MecA present reveal the interaction of this MD phenylalanine residue with MecA such that intermolecular MD-MD interactions are disrupted upon introduction of MecA, thereby reconfiguring the complex into an enzymatically active hexamer. However, in the absence of an intact Uvr motif, the inactive helical assembly is no longer stabilized via MD-MD intermolecular interactions and MecA is no longer necessary for ClpC activation.

**Figure 6:**
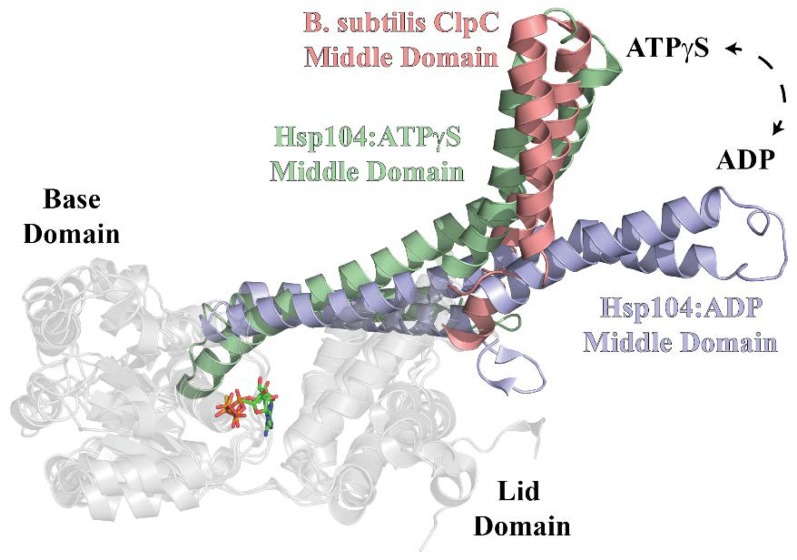
Structural Comparisons Among Middle Domain-Containing Hsp100 proteins. Alignment of X-ray structures for Hsp104 bound to either ATPγS (green) or ADP (purple) alongside *B. subtilis* ClpC (salmon) reveal the Middle Domain conformation to be nucleotide-dependent. The PDB accession codes used to create Figure 6 were 5VY9, 5VY9 and 3PXG.

Cyro-EM structures for *S. cerevisiae* Hsp104 have led to the proposal that the MD adopts two nucleotide-specific conformations that correspond to the hydrolytic state of the bound nucleotide (Figure 6) [33]. From this, the MD was proposed to stabilize the ATP-state of Hsp104 subunits with bound polypeptide substrate. Superimposition of the X-ray structure for *B. subtilis* ClpC-D1 [31] alongside the Cryo-EM structures for Hsp104 [33] bound to either ATPγS or ADP positions the ClpC-MD in an ATP-like conformation that is approximately orthogonal to the position predicted for the ADP-state (Figure 6). Unlike the Hsp104 structures, however, the model shown for ClpC is derived from a co-crystal structure with MecA bound, which suggests a role for adaptor protein in stabilization of the ATP-state. 

Our data presented here suggest the presence of two distinct ClpC1 sites that participate in ClpS interactions. Based on pulldown data and comparisons with homologous Clp/Hsp100 ATPases, those sites are represented by the ClpC1-NTD and ClpC1-MD. Though the ClpC1-NTD is dispensable for ClpC1 catalyzed unfolding of an SsrA-tagged protein, it may contribute to complex stability. However, our molecular dynamics model indicates that the ClpS:ClpC1-NTD interaction may differ substantially from the models reported for the ClpA-NTD:ClpS complex [10,36]. In our model, much of the free energy associated with complex formation involves a single interaction between Lys85 of ClpC1-NTD and Asp158 of ClpS. Though an Asp is conserved at this position in all ClpS proteins, reference to comparable structures for ClpA-NTD:ClpS complexes indicate that this residue is positioned on the ClpS-face opposite to the ClpA-interaction site [10,36]. Furthermore, no interaction pair identified in our ClpC1-NTD:ClpS model would be expected to occur similar to those reported for ClpA. However, our stopped-flow fluorescence data represented in Figure 2 and Figure 3 demonstrate that the ∆NTD-ClpC1 truncation mutant is subject to allosteric inhibition by ClpS, which requires that a physical interaction occur between the two proteins in the absence of an intact ClpC1-NTD. Further mutations to residue positions surrounding the Uvr motif, D440A and R451A, ablate ClpS-dependent inhibition of ClpC1 catalyzed SsrA-tagged protein unfolding. Taken together, our data support a model wherein ClpS interacts directly with the ClpC1-NTD and -MD in a configuration that may be more “MecA-like.” Based on the structural data presented in Figure 6, we expect that this would necessarily need to involve the ClpS-dependent stabilization of a ClpC1-MD conformation other than the ATP-state. However, the structural details of these conformational dynamics are currently unclear.

Taken together, our data suggest that ClpS functions as binary regulator of ClpC1 catalyzed protein unfolding (Figure 1C). This statement is based on the observation in Figure 1C of two phases separated by a narrow range of [ClpS], which we term the Catalytic and Partially-Inhibited phases (colored orange and yellow in Figure 1C, respectively). The average apparent unfolding rates are 1.7 ± 0.2 or 0.8 ± 0.1 s^−1^ in the catalytic state or partially-inhibited state, respectively. This contrasts reports involving *E. coli* ClpAP catalyzed degradation of SsrA-tagged GFP in the presence of ClpS, where a continuum of kinetic behaviors are observed such that inhibition of ClpAP catalyzed protein degradation occurs over a broader range of [ClpS] [11]. 

Though Clp/Hsp100 proteins are highly homologous in primary sequence and overall architecture, regulation of this family of proteins is not necessarily identical across all bacterial species. The data reported in this study illustrate that regulation of mycobacterial ClpC proteins cannot be assumed as functionally equivalent to well-studied Clp/Hsp100 proteins such as *E. coli* ClpA. We demonstrate that ClpS-dependent inhibition of *M. tuberculosis* ClpC1 catalyzed unfolding of SsrA-proteins does not require an intact ClpC1-NTD and involves the ClpC1-MD. This contrasts reported data for *E. coli* ClpA, where the ClpA-NTD is necessary for the observation of ClpS-dependent inhibition of function. Our data qualitatively may suggest a role for the ClpC1-NTD in stabilization of the ClpC1:ClpS complex. This observation is of potential clinical significance since anti-tuberculosis drugs such as cyclomarin A [30,37] and lassomycin [38,39] are expected to bind to the ClpC1-NTD, thereby leading to competition for binding between ClpS and anti-tuberculosis drug. However, future work will be needed to clarify whether ClpS-overexpression in mycobacteria represents a viable resistance mechanism against these novel anti-tuberculosis drugs. 

## 4. Materials and Methods

### 4.1. Materials

All solutions were prepared with reagent-grade chemicals in double-distilled water produced from a Purelab Ultra Genetic System (Siemens Water Technology, Munich, Germany). All genes were synthesized and each cloned into the pET-24a(+) vector commercially by Genscript (Piscataway, NJ, USA). Plasmids for ClpC1 proteins encode His_6_-SUMO fusions with full-length ClpC1, ClpC1 truncation mutant lacking N-terminal residues M1-Y145 (∆NTD-ClpC1), the isolated ClpC1 N-terminal domain (ClpC1-NTD), or ∆NTD-ClpC1 bearing two single-point mutations, D440A/R451A, in the ClpC1-MD (∆NTD-ClpC1-D440A/R451A).

### 4.2. Protein Expression and Purification

All expression constructs were prepared as N-terminal His_6_-SUMO fusions and overexpressed from the pET-24a(+) vector in BL21(DE3) competent cells. Bacterial cultures were initially grown in Lysogeny broth (LB) at 37 °C, followed by induction at OD_600_ = 0.6 absorbance units with 0.5 mM isopropyl β-d-1-thiogalactopyranoside (IPTG, ThermoFisher Scientific, Waltham, MA, USA) and overnight incubation with shaking at 18 °C. The harvested cell paste was resuspended in chilled lysis buffer containing 50 mM Tris (pH = 8.3), 400 mM NaCl, 10% glycerol, 1 mM 2-mercaptoethanol, 10 mM imidazole (pH = 8) and a protease inhibitor cocktail tablet (ThermoFisher Scientific). The resulting suspension was subjected to sonication and clarified by centrifugation at ~50,000× *g*. Affinity chromatography was next applied such that the supernatant resulting from the previous centrifugation step was incubated at 4 °C for 2 h with Ni-nitriloacetic acid solid-phase resin (Ni-NTA, G-Biosciences, St. Louis, MO, USA) previously equilibrated with lysis buffer. Following incubation, the Ni-NTA resin was subjected to centrifugation at ~250× *g* to isolate solid-phase resin. The supernatant was discarded and the Ni-NTA resin washed with fresh lysis buffer. This wash cycle was repeated five to seven times in order to remove any proteins not associated with the Ni-NTA resin. After the final Ni-NTA wash cycle, all His_6_-SUMO-fusion proteins were dissociated from the resin by gravity flow using elution buffer containing 50 mM Tris pH = 8.3, 300 mM NaCl, 10% glycerol, 2 mM 2-mercaptoethanol and 500 mM imidazole (pH = 8). Unless otherwise stated, the His_6_-SUMO tag was removed from all fusion proteins by overnight digestion with His_6_-tagged Ulp1 protease in elution buffer [40]. Cleaved protein was separated from His_6_-Ulp1 and any residual uncleaved protein by a second round of Ni-NTA binding. For *M. tuberculosis* His_6_-SUMO-ClpS, His_6_-SUMO-ClpC1-NTD and *T. geoffroyi* His_6_-SUMO-SsrA-Kaede (expressed with C-terminal *M. tuberculosis* SsrA sequence, AADSHQRDYALAA [19]) fusion proteins, the resulting flow-through volume was dialyzed overnight against H200 buffer (25 mM HEPES (4-(2-hydroxyethyl)-1-piperazineethanesulfonic acid) (pH = 7.6), 200 mM NaCl, 10 mM MgCl_2_, 1 mM 2-mercaptoethanol and 10% glycerol), flash-frozen in liquid nitrogen and stored at −80 °C.

Additional purification steps were applied to isolate full-length ClpC1, ∆NTD-ClpC1 and ∆NTD-DR440AA. After removal of the His_6_-SUMO tag, the resulting cleaved ClpC1 protein solution was diluted with lysis buffer lacking NaCl to a final [NaCl] = 100 mM and loaded onto a Hiprep Q FF 16/10 column (GE Healthcare, Piscataway, NJ, USA) previously equilibrated with 20 mM Tris (pH = 8.3), 10 mM NaCl, 1 mM 2-mercaptoethanol and 10% glycerol. The sample was eluted with a linear gradient from 10 mM NaCl to 1000 mM NaCl over 8 column volumes. Fractions derived from the linear gradient elution were subjected to analysis by SDS-PAGE to confirm the presence of ClpC1 protein and pooled accordingly. Pooled fractions were dialyzed overnight against storage buffer containing (50 mM Tris pH = 8.3, 400 mM NaCl, 5 mM 2-mercaptoethanol and 50% glycerol), flash-frozen in liquid nitrogen and stored at −80 °C. Prior to storage, purity was judged to be >95% by Coomassie staining (Figure S2A).

Protein concentrations were determined spectrophotometrically in reaction buffer H200 using extinction coefficients ε_280_ = 3.59 × 10^4^ M^−1^·cm^−1^, ε_280_ = 3.14 × 10^4^ M^−1^·cm^−1^, ε_280_ = 2.65 × 10^4^ M^−1^·cm^−1^, ε_280_ = 4.47 × 10^3^ M^−1^·cm^−1^ and ε_280_ = 2.74 × 10^4^ M^−1^·cm^−1^, respectively, for full-length *M. tuberculosis* ClpC1, ∆NTD-ClpC1/∆NTD-ClpC1-D440A/R451A, *M. tuberculosis* ClpS, ClpC1-NTD and *T. geoffroyi* SsrA-Kaede. The concentration of His_6_-SUMO-ClpS was determined spectrophotometrically in H200 buffer described above using an extinction coefficient of ε_280_ = 2.79 × 10^4^ M^−1^·cm^−1^. *T. geoffroyi* SsrA-Kaede was subjected to photoactivation prior to storage using methods previously described [23,24,25].

### 4.3. Methods

#### 4.3.1. Stopped-Flow Fluorescence Assay

Stopped-flow fluorescence experiments were performed using an Applied Photophysics SX.20 stopped-flow fluorometer (Letherhead, UK). All reactions were performed at 37 °C in buffer H200 (25 mM HEPES (pH = 7.6), 200 mM NaCl, 10 mM MgCl_2_, 1 mM 2-mercaptoethanol and 10% (*v*/*v*) glycerol). Syringe A contained 1 µM ClpC1 and varied initial concentrations of ClpS from 0 to 3 µM. Syringe B contained 100 nM of photoactivated Kaede (SsrA-Kaede_Red_) bearing a C-terminal *M. tuberculosis* SsrA degradation tag and 9.5 mM ATP. The observation of ClpC1 catalyzed protein unfolding requires the inclusion of ATP in Syringe B (Figure S3). Prior to mixing, both solutions were incubated for 15 min at 37 °C in the stopped-flow instrument to establish thermal equilibrium. Additional incubation of either solution had no effect on the observed fluorescence time courses. SsrA-Kaede_Red_ was excited at λ_ex_ = 568 nm and emissions were observed above 570 nm using a 570-nm-long pass filter. All kinetic traces shown represent the average of at least seven individual determinations. Averaged time courses were subjected to non-linear least squares (NLLS) analysis using a single exponential function. The dependence of the apparent unfolding rate constant, *k_UF,app_*, on the final mixing concentration of ClpS, [ClpS]_F_, was subjected to NLLS analysis using a modified form of the Hill equation:

Equation (1)

(1) $$k_{UF,\;app}=1-\left(k_{max}\cdot \frac{(K_{app}{\left[ClpS{]}_{F}\right)}^{n}}{1+(K_{app}{\left[ClpS{]}_{F}\right)}^{n}}+b\right)$$

where *k_max_* represents the maximum apparent unfolding rate constant, *K_app_* approximates the association equilibrium constant, *n* is the Hill coefficient and *b* is the *y*-intercept term.

#### 4.3.2. Ni-NTA Pulldown Experiments

Ni-NTA affinity pulldown experiments were performed by incubating 2 µM His_6_-SUMO-ClpS with varied concentrations of ClpC1 and 1 mM ATPγS (as indicated) at 25 °C for 30 min to promote complex formation. All experiments were performed in 150 µL reaction volumes in buffer H200 (25 mM HEPES (pH = 7.6), 200 mM NaCl, 10 mM MgCl_2_, 1 mM 2-mercaptoethanol and 10% glycerol). After initial incubation, 100 µL of Ni-NTA slurry, in H200 buffer supplemented with 1 mM ATPγS and 10 mM imidazole (pH = 8), was added to each reaction solution, followed by incubation with agitation at 25 °C for 60 min to promote Ni-NTA binding by His_6_-SUMO-ClpS. After 60 min, each reaction mixture was transferred to an empty polypropylene column (Bio-Rad Laboratories, Hercules, CA, USA), where solid resin was isolated from buffer by gravity flow. The isolated Ni-NTA resin was washed 3 times with an excess volume of H200 supplemented with 1 mM ATPγS and 10 mM imidazole (pH = 8). His_6_-SUMO-ClpS was eluted from the Ni-NTA resin by the addition of 200 µL of H200 supplemented with 1 mM ATPγS and 250 mM imidazole (pH = 8). The resulting elution samples were analyzed by SDS-PAGE using either Coomassie- or silver-staining methods to visual gel bands.

#### 4.3.3. Structure Preparation

The PDB file for the crystal structure of ClpC1 [30] was obtained from the Protein Data Bank [41] (PDB ID: 3WDB) and included an expression tag at N-terminus. The three-dimensional structure of *M. tuberculosis* ClpS was obtained from homology modeling with the SWISS-Model Server [42] using the crystal structure for ClpS from *E. coli* [43] from the protein databank (PDB ID: 2WA9) as a template.

The 3D structures for ClpC1-NTD and ClpS from *M. tuberculosis* were prepared for docking using the MOE2018 [44] software suite (2018, Montreal, QC, Canada). MOE’s Protonate 3D [45] utility was used to add the appropriate amount of hydrogens to each structure at a pH of 7.5, salt concentration of 0.15M and temperature of 310 K. The protonated structures were energy minimized using the AMBER14:EHT [46,47] force field.

#### 4.3.4. Docking Process

The prepared structures of *M. tuberculosis* ClpC1-NTD and ClpS were submitted to the ClusPro 3.0 [48] server for rigid body docking calculations. The structure for ClpC1 was defined as the receptor and that of *M. tuberculosis* ClpS was defined as the ligand. No residues were designated to be attractive or repulsive. 

#### 4.3.5. Molecular Dynamics

Molecular dynamics simulations were performed using the NAMD2 platform [49]. The initial structure was from the docking result with the lowest binding energy. Explicit solvent was employed using the TIP3P water model [50]. The NPT ensemble was used with a constant temperature of 310 K. The damping coefficient used in the simulations was set as 5 ps. The MD pressure was set as 1 atm and kept as constant with Langevin piston method [51,52]. In the simulations, no constraint was applied to any atomic coordinates. To include long-range electrostatic interactions in the simulations, the particle-mesh Ewald (PME) method was used with a 1-angstrom grid width [53]. The nonbonded interactions were evaluated every 10 time-steps using a group-based cutoff with a switching function. The SHAKE algorithm [54] was employed to fix the covalent bonds involving hydrogen in the simulations [54]. The systems were equilibrated for 20 ns, followed by another 20 ns molecular dynamics runs. The last snapshot was selected used for the binding analysis. 

## Figures and Tables

**Table 1:** Apparent rate constant describing ClpC1 catalyzed SsrA-Kaede_Red_ unfolding as a function of [ClpS]_F_.

[ClpS]_F_ (nM)	*k_UF,app_* (s^−1^)	
	Full-Length ClpC1	∆NTD-ClpC1
0	1.7 ± 0.1	2.4 ± 1.1
125	1.4 ± 0.1	
250	1.9 ± 0.3	1.4 ± 0.1
350	0.9 ± 0.1	
500	0.9 ± 0.1	
1000	0.7 ± 0.3	0.9 ± 0.2
1500	0.89 ± 0.03	

*K_UF,app_* is the apparent rate constant describing ClpC1 catalyzed unfolding of an SsrA-tagged protein. [ClpS]_F_ represents the final reaction concentration of ClpS after mixing in the stopped-flow spectrophotometer.

**Table 2:** List of the contact residues. The distance and interaction energy is in unit of Å and kcal/mol.

ClpC1	ClpS	Energy	Dist
Phe2	Trp33	−4.802	3.95
Phe2	Asp34	−0.118	4.26
Phe2	Trp94	−2.193	3.95
Phe2	Thr96	−0.877	4.19
Glu3	Thr96	−1.604	4.16
Thr6	Arg101	−0.128	4.21
Asp7	Trp33	−0.252	3.87
Asp7	Gln98	−3.051	3.99
Asp7	Arg101	0.018	4.20
Arg10	Trp33	−5.015	3.93
Arg10	Gln98	−1.263	3.52
Val13	Asp35	−0.257	4.34
Val13	Pro36	−1.65	4.09
Val14	Glu68	−0.466	4.35
Val14	Gly69	−0.605	4.02
Gln17	Pro36	1.15	4.19
Gly76	His66	−0.244	3.89
Gly76	Asn67	0.098	3.92
His77	Pro36	1.52	3.94
His77	Val37	0.391	4.21
His77	Asn38	−0.334	4.02
His77	Leu39	0.291	4.29
His77	His66	1.173	3.83
Ile78	Pro36	−0.065	3.94
Pro79	Val37	−0.877	3.71
Pro79	Asn38	0.5	4.47
Phe80	Pro36	−1.774	4.02
Phe80	Val37	−2.005	3.99
Lys85	Asp35	−22.528	3.47
Lys85	Pro36	−7.72	3.62
Lys85	Val37	−1.107	4.02
Tyr145	Arg101	−0.077	3.98

## Supplementary Materials

Supplementary materials can be found at http://www.mdpi.com/1422-0067/19/11/3651/s1.

## Abbreviations

NTD	N-terminal domain
MD	Middle domain
NTP	Nucleoside triphosphate
ATP	Adenosine triphosphate
AAA+	ATPases associated with various cellular activities
