# Supplementary material for: Mycobacterium tuberculosis ClpC1 N-Terminal Domain Is Dispensable for Adaptor Protein-Dependent Allosteric Regulation
Source: Int J Mol Sci. 2018 Nov 19;19(11):3651. doi: 10.3390/ijms19113651 (PMC6274998; doi:10.3390/ijms19113651)

# Supplemental Material

## Figure S1.

***Full-length ClpC1 Interacts Non-Specifically with Ni-NTA Resin***. A) Control experiments performed as described in **Figure 4** reveal non-specific binding between Ni-NTA and full-length ClpC1 in the absence of His$_6$-SUMO-ClpS. B) Identical experiments performed with ΔNTD-ClpC1 do not support the same conclusion in the absence of an intact N-terminal domain. All SDS-PAGE gels shown in Figure S1 have been visualized by silver staining methods.

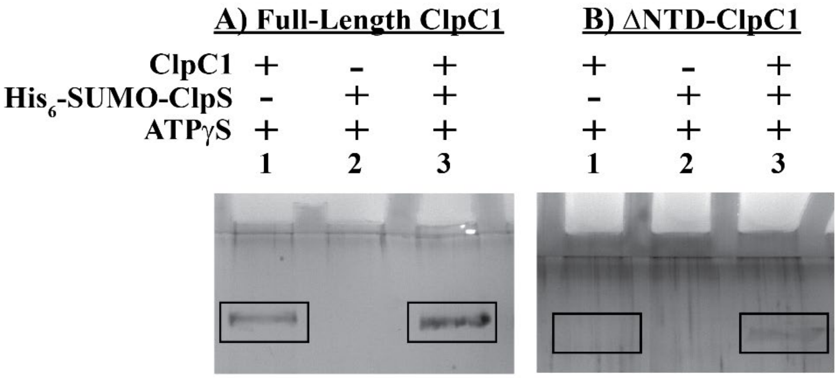


## Figure S2.

***M. tuberculosis ClpC1 Purification***. A) Active ClpC1 can be isolated to greater than 95% purity as judged by Coomassie staining. Lanes on a 10 % acrylamide gel were loaded as follows: 1 – molecular weight standard, 2 – soluble lysate, 3 – Supernatant from Ni-NTA wash, 4 – Ni-NTA flow-through solution, 5 – Ni-NTA elution, and 6 – pooled fractions after His$_6$-Ulp1 cleavage and ion exchange chromatography. B) Size-exclusion chromatography analysis has been performed as a final purification step in place of ion exchange chromatography. Fractions containing ClpC1 elute at a volume that approximately corresponds to the mass of a dimer.

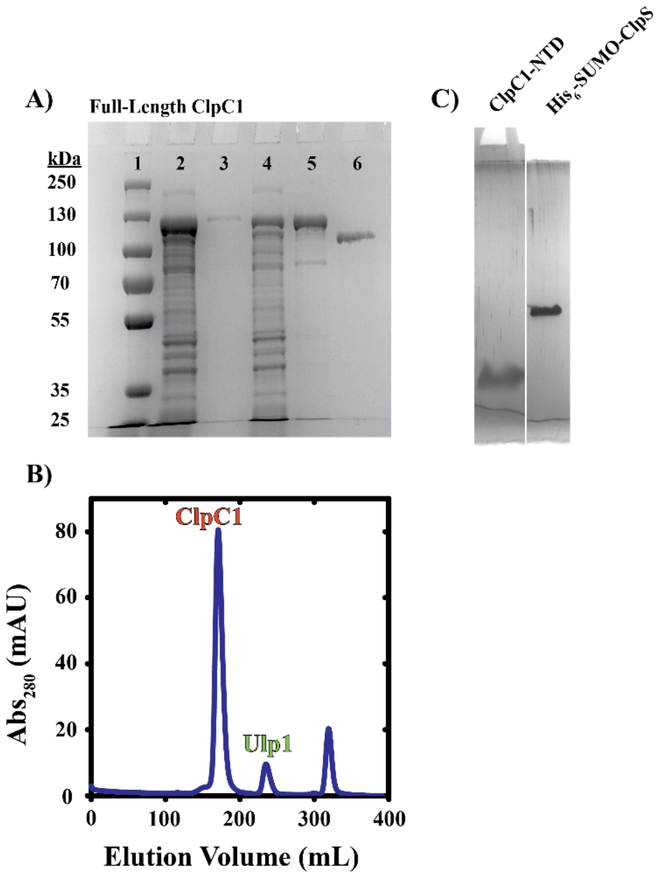


## Figure S3.

***ClpC1 catalyzed protein unfolding requires ATP.*** A) Schematic representation of stopped-flow fluorescence protein unfolding experiments. Syringe A contains 1 μM ClpC1. Syringe B contains 9.5 mM ATP to fuel protein unfolding and 100 nM photoactivated Kaede bearing a C-terminal SsrA-degradation tag (SsrA-Kaede$_{Red}$). Fluorescence is observed using an excitation wavelength equal to 568 nm and emissions are observed above 570 nm with a 570-nm-long pass filter. B) Representative fluorescence time courses for ClpC1 catalyzed SsrA-KaedeRed unfolding. Time courses were measured in the presence (Blue Circles) or absence (Red Circles) of 9.5 mM ATP.

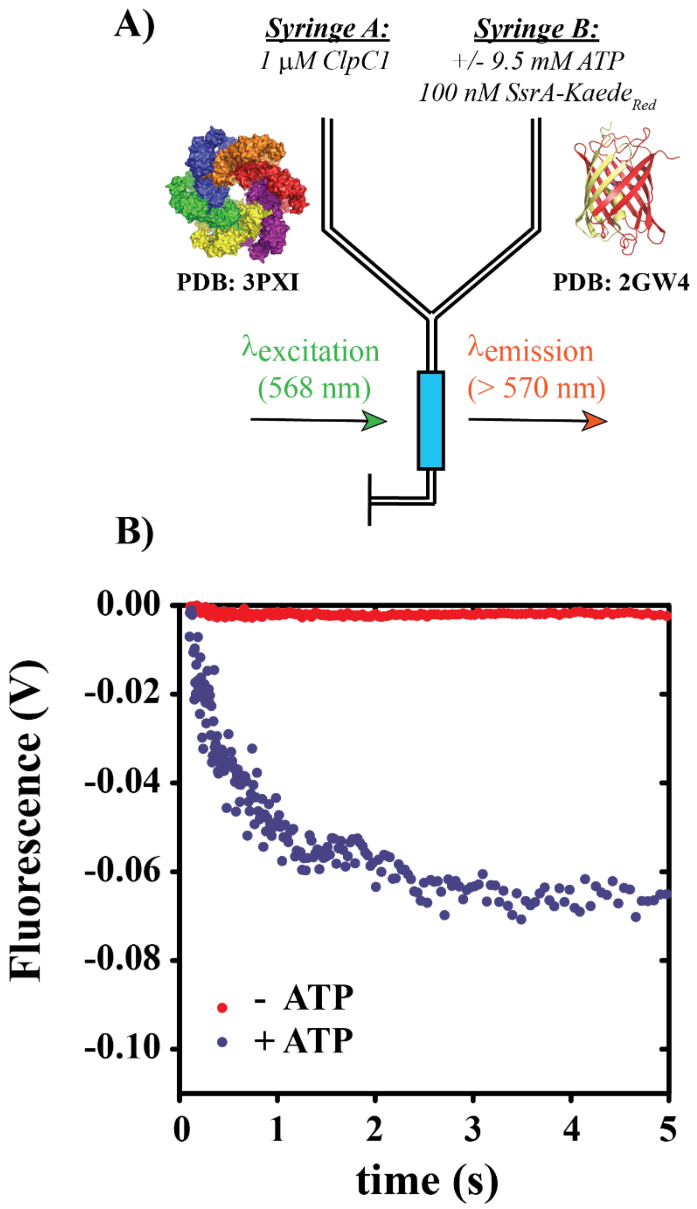

Supplement: Supplementary file 1 [file ijms-19-03651-s001.pdf]
